# Water mass age structures the auxiliary metabolic gene content of free-living and particle-attached deep ocean viral communities

**DOI:** 10.1186/s40168-023-01547-5

**Published:** 2023-05-27

**Authors:** Felipe H. Coutinho, Cynthia B. Silveira, Marta Sebastián, Pablo Sánchez, Carlos M. Duarte, Dolors Vaqué, Josep M. Gasol, Silvia G. Acinas

**Affiliations:** 1 Department of Marine Biology and Oceanography, Institut de Ciències del Mar (ICM), CSIC, 08003 Barcelona, Spain; 2 Department of Biology, University of Miami, Coral Gables, FL USA; 3 Department of Marine Biology and Ecology, Rosenstiel School of Marine, Atmospheric, and Earth Sciences, University of Miami, Miami, FL USA; 4 Red Sea Research Centre (RSRC) and Computational Bioscience Research Center (CBRC), King Abdullah University of Science and Technology, Thuwal, 23955 Saudi Arabia

**Keywords:** Viruses, Metagenomics, Deep ocean, Auxiliary metabolic genes, Free-living, Particle-attached

## Abstract

**Background:**

Viruses play important roles in the ocean’s biogeochemical cycles. Yet, deep ocean viruses are one of the most under-explored fractions of the global biosphere. Little is known about the environmental factors that control the composition and functioning of their communities or how they interact with their free-living or particle-attached microbial hosts.

**Results:**

We analysed 58 viral communities associated with size-fractionated free-living (0.2–0.8 μm) and particle-attached (0.8–20 μm) cellular metagenomes from bathypelagic (2150–4018 m deep) microbiomes obtained during the Malaspina expedition. These metagenomes yielded 6631 viral sequences, 91% of which were novel, and 67 represented high-quality genomes. Taxonomic classification assigned 53% of the viral sequences to families of tailed viruses from the order Caudovirales. Computational host prediction associated 886 viral sequences to dominant members of the deep ocean microbiome, such as Alphaproteobacteria (284), Gammaproteobacteria (241), SAR324 (23), Marinisomatota (39), and Chloroflexota (61). Free-living and particle-attached viral communities had markedly distinct taxonomic composition, host prevalence, and auxiliary metabolic gene content, which led to the discovery of novel viral-encoded metabolic genes involved in the folate and nucleotide metabolisms. Water mass age emerged as an important factor driving viral community composition. We postulated this was due to changes in quality and concentration of dissolved organic matter acting on the host communities, leading to an increase of viral auxiliary metabolic genes associated with energy metabolism among older water masses.

**Conclusions:**

These results shed light on the mechanisms by which environmental gradients of deep ocean ecosystems structure the composition and functioning of free-living and particle-attached viral communities.

Video Abstract

**Supplementary Information:**

The online version contains supplementary material available at 10.1186/s40168-023-01547-5.

## Background

Aquatic viruses play major roles in the structuring of microbiomes and modulate biogeochemical cycles of global relevance [1, 2]⁠. Global scale studies based on viral metagenomics have brought significant advances to our understanding of viral genomic diversity and the environmental factors that structure their communities [3–5]⁠. These studies most often evaluated viruses from the epipelagic and mesopelagic zones, while the bathypelagic received less attention, although some notable exceptions led to significant advances in the field [6–9]⁠. Nevertheless, our understanding of viral diversity in the largest marine ecosystem, the deep ocean, is still limited [6, 7, 10]⁠. Compared to the epipelagic zone, the bathypelagic is characterised by the absence of light, low temperatures, very low concentrations of labile carbon, and higher concentrations of inorganic nutrients [11]⁠. Also, the bathypelagic has lower densities of prokaryotic cells and viral particles but higher virus-to-prokaryote ratios [11–13]⁠. In the bathypelagic, both free-living and particle-attached microbial communities are active but differ in taxonomic and functional composition, cell densities, and activity levels [14, 15]⁠. These differences affect the structure and functioning of the viral communities that infect them [8]⁠. Furthermore, evidence suggests that deep ocean viruses contribute to organic matter remineralisation through lysis of particle-attached heterotrophic hosts, possibly enhancing carbon export efficiency [16]⁠. Yet, there is little information regarding which are the hosts targeted by these viruses, their genetic diversity, and the mechanisms by which they interact with their host communities.

Viral genomes often encode auxiliary metabolic genes (AMGs) that redirect host metabolism towards pathways that benefit viral replication during infection [4, 17]⁠. Changes in host metabolism mediated by the expression of AMGs impact global element and energy cycles [18–20]⁠. The deep ocean has a unique set of AMGs [21–23]. Yet, the physical and chemical parameters driving the composition of this genetic repertoire in deep ocean water masses have not been assessed. Likewise, the differences in viral taxonomy, host prevalence, and AMG content between viruses associated with the free-living and particle-attached communities have not been explored in detail.

The Malaspina expedition has contributed to the description of microbial diversity and functioning in the oceans [14, 24]⁠. Findings derived from this expedition revealed that deep ocean basins and water masses have a major role in structuring the composition of bathypelagic communities of bacteria, archaea, and micro-eukaryotes [24, 25]⁠. Bathypelagic free-living (FL) and particle-attached (PA) communities have markedly distinct taxonomic [24] and functional compositions [14]⁠. While FL microbial assemblages are more diverse and contain more oligotrophic taxa, the PA microbial assemblages are less diverse and contain more copiotrophic taxa [24]⁠, although some taxa occur in both fractions, displaying a dual lifestyle [15]⁠. Similarly, viral community diversity is different across viral and cellular size fractions [6, 20]⁠. Most studies of bathypelagic viruses have focused on free viral particles in the smallest size fraction (< 0.22 μm) [7, 9, 10]⁠. Less is known regarding the environmental parameters that control the structure and functioning of the viral communities in the cellular fraction (> 0.22 μm), i.e. those associated with particle-attached or free-living host communities. Based on previous findings, we hypothesise that bathypelagic free-living and particle-attached viral communities differ as a consequence of the differences in host community composition and functioning. We postulate that particle-attached communities are enriched in viruses that target copiotrophic bacteria, as particles are considered resource-rich micro-environments within the bathypelagic, where labile organic matter sources are depleted. We further hypothesise that the environmental parameters that characterise different water masses in the deep ocean also shape the taxonomic composition and AMG content of bathypelagic viral communities, specifically regarding metabolic pathways associated with energy and resource availability.

## Results and discussion

### Novel viruses from the bathypelagic zone have a unique genetic repertoire

**Fig. 1:**
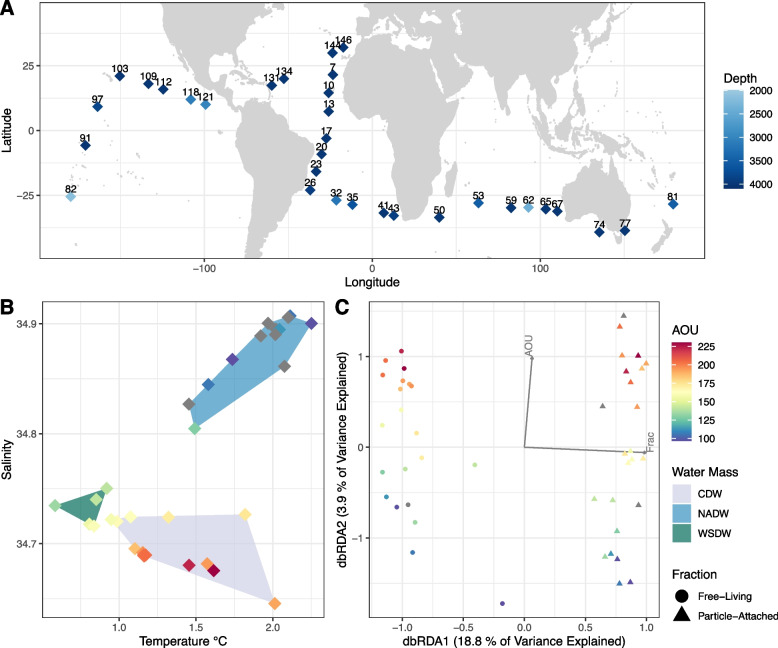
Global trends of the deep ocean viral communities. **A** World map displaying sampling sites from which Malaspina bathypelagic metagenomes were retrieved. **B** Scatter plot displaying the values of salinity, temperature, and AOU measured at the sites from which metagenome samples were retrieved. **C** dbRDA biplot constrained by size fraction and AOU

The Malaspina samples represent the tropical and subtropical bathypelagic ocean (Fig. 1A and Table S1). Three main water masses were identified, which differed according to salinity, temperature, and apparent oxygen utilisation (AOU): circumpolar deep water (CDW), North Atlantic Deep Water (NADW), and Weddell sea deep water (WSDW) [26], (Fig. 1B). Cellular metagenomes were generated from 28 samples from the free-living (0.2–0.8 μm) fraction and 30 samples from the particle-attached (0.8–20 μm) fraction, for a total of 58 metagenomes (Table S1). Out of 422,928 scaffolds derived from the co-assembly of the metagenomes, VIBRANT [27] classified 6631 scaffolds as fully viral (6479) or as viral fragments (152) within longer scaffolds (Table S2). Among these, CheckV [28] categorised 23 as complete genomes and 44 as high-quality genome fragments (i.e. estimated completeness ≥ 90%). VPF-Class [29] classified 5100 scaffolds as dsDNA viruses and 33 as ssDNA viruses. The most common families were Myoviridae (1856), Siphoviridae (1039), Podoviridae (637), and Phycodnaviridae (86). The most common taxa assigned as putative hosts of the viral scaffolds by PHIST [30]⁠ were Alphaproteobacteria (284), Gammaproteobacteria (241), Chloroflexota (61), Bacteroidota (60), and Marinisomatota (39).

The 563,348 coding DNA sequences (CDS) derived from the viral genomes and genome fragments were annotated against the UniRef, Pfam, and KEGG databases (Table S3). All CDS were also queried against a custom reference database of isolated and uncultured viral genomes encompassing multiple ecosystems [31]⁠. Pairwise similarity was calculated between Malaspina viral scaffolds and reference database genomes, based on the percentage of matched CDS. Only 598 (9%) of the Malaspina viral scaffolds shared 10 or more CDS with a single sequence in the reference database. This threshold is far below the ICTV defined cutoffs of average nucleotide identity (ANI) for species (≥ 95%) and genera (≥ 70%) established for most taxa of dsDNA viruses that infect Archaea or Bacteria [32]. Thus, these results suggest that 91% of Malaspina viral sequences are novel, while the remaining ones only have distant relatives in reference databases.

### Free-living and particle-attached metagenomes have unique viral communities

Viral scaffold relative abundances (reads per kilobase per million reads, RPKM) were calculated by read mapping to determine the viral community composition in the metagenomes (Table S4). To investigate how AOU (which is a proxy for water mass age) and size fraction influence the composition of viral communities, we performed distance-based redundancy analysis (dbRDA) using the aforementioned parameters as constraining variables (Fig. 1C). Unconstrained axes explained 77.2% of the total variance, while constrained axes explained 22.7% of the total variance. Specifically, dbRDA1 explained 4.27% of the total variance (18.8% of the constrained variance), while dbRDA2 explained 0.89% of the total variance (3.9% of the constrained variance). PERMANOVA pointed to significant influences of size fraction (*p* < 0.001) and AOU (*p* < 0.05) on viral community composition. The AOU variable integrates all respiratory processes since water mass formation [33]⁠; thus, older water masses display higher values of apparent oxygen utilisation. The AOU of the three water masses differed according to their age: NADW (median age = 481 years, median *AOU* = 107 μmol kg^−1^), WSDW (median age = 545 years, median *AOU* = 142 μmol kg^−1^), and CDW (median age = 1046 years, median AOU = 193 μmol kg^−1^). In addition, we performed Mantel tests to detect significant associations between environmental parameters and community composition. Euclidean distances derived from O_2_ concentrations, which strongly correlated with AOU in our dataset, displayed the strongest association with Bray-Curtis distances derived from viral scaffold relative abundances (Mantel statistic = 0.11, *p*-value = 0.009). Together, these results corroborated our claim that size-fraction and water mass age are the variables that best explained differences in viral community composition among these metagenomes. Apparent oxygen utilisation had previously been shown to be an important factor associated with host community composition in the bathypelagic [15, 24]⁠. Our results corroborate these findings and extend them by showing that water mass age is a significant driver of bathypelagic viral community composition.

**Fig. 2:**
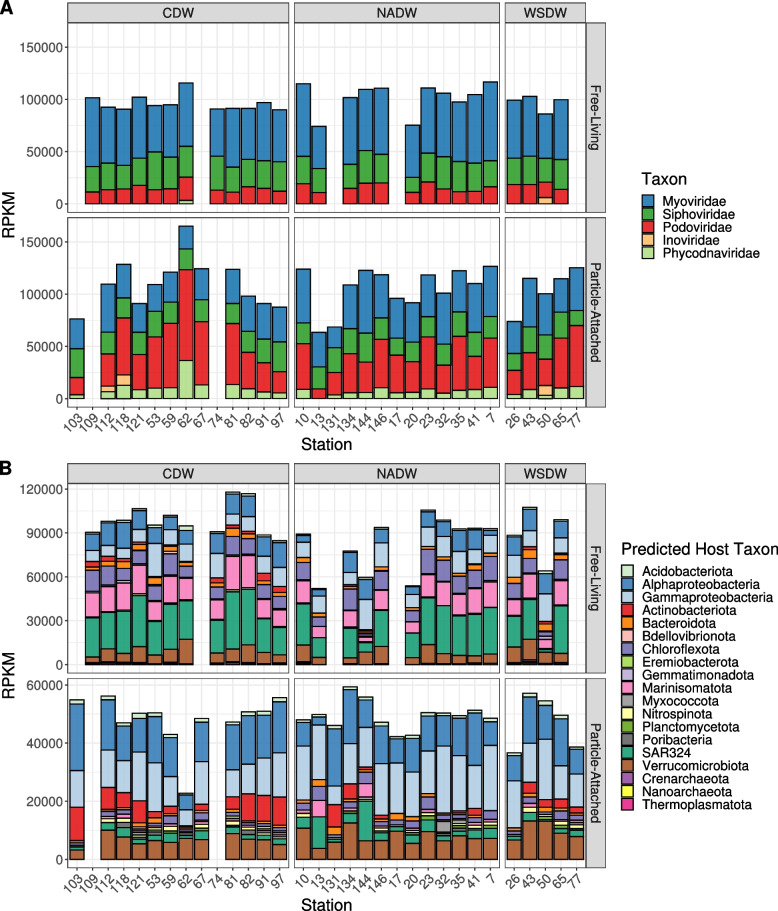
Differences in viral community taxonomic composition between free-living and particle-attached samples. **A** RPKM abundances of viral genomes grouped by family level taxonomic classification. **B** RPKM abundances of viral genomes grouped by predicted host phylum (or class for Proteobacteria). Sampling stations are sorted from left to right by increasing oxygen concentrations. Sample MP0262 was excluded from this analysis as it displayed extremely high RPKM values, not in line with those observed in any other samples

We next analysed how viral community composition shifted between free-living and particle-attached fractions. We calculated the relative abundances of viruses grouped by taxonomic family (Table S8). Families Myoviridae, Siphoviridae, and Podoviridae were the dominant taxa across all samples, regardless of fraction or water mass (Fig. 2A). Yet, the families Myoviridae and Siphoviridae were significantly more abundant among FL samples (Mann-Whitney test, *p* < 0.01), while families Podoviridae and Phycodnaviridae were significantly more abundant among PA samples (Mann-Whitney test, *p* < 0.01). Phycodnaviridae only had abundances above 2500 RPKM in the particle-attached fraction (except for station 62). Viruses of the family Phycodnaviridae infect eukaryotic microalgae, hence their higher abundance among samples from the larger size fraction, as sinking particles may be composed of phytodetritus [34] or even intact phytoplankton cells [35].

We calculated the relative abundances of viruses grouped by predicted host phylum (or class for Proteobacteria, Table S9). Viruses predicted to infect Alphaproteobacteria and Gammaproteobacteria were among the most abundant ones in both PA and FL samples (Fig. 2B). Nevertheless, multiple viral groups displayed significantly different relative abundances among PA and FL samples (Mann-Whitney test, *p* < 0.01). Viruses predicted to infect Marinisomatota, Chloroflexota, and SAR324 were more abundant among FL samples, while viruses predicted to infect Myxococcota, Planctomycetota, and Actinobacteriota were more abundant among PA samples. These trends are in agreement with the previously observed taxonomic composition of the host communities reported for the same samples [14, 15, 24, 36]⁠. We postulate that differences in target host prevalence between the two fractions are a consequence of differences in the preferred ecological niche of deep ocean microbes, which can be roughly divided into particle-attached copiotrophs (e.g. Gammaproteobacteria and Actinobacteriota), free-living oligotrophs (e.g. SAR324 and Chloroflexota), and those that present a dual lifestyle [15]⁠.

### Viral auxiliary metabolic gene content shifts between free-living and particle-attached fractions

**Fig. 3:**
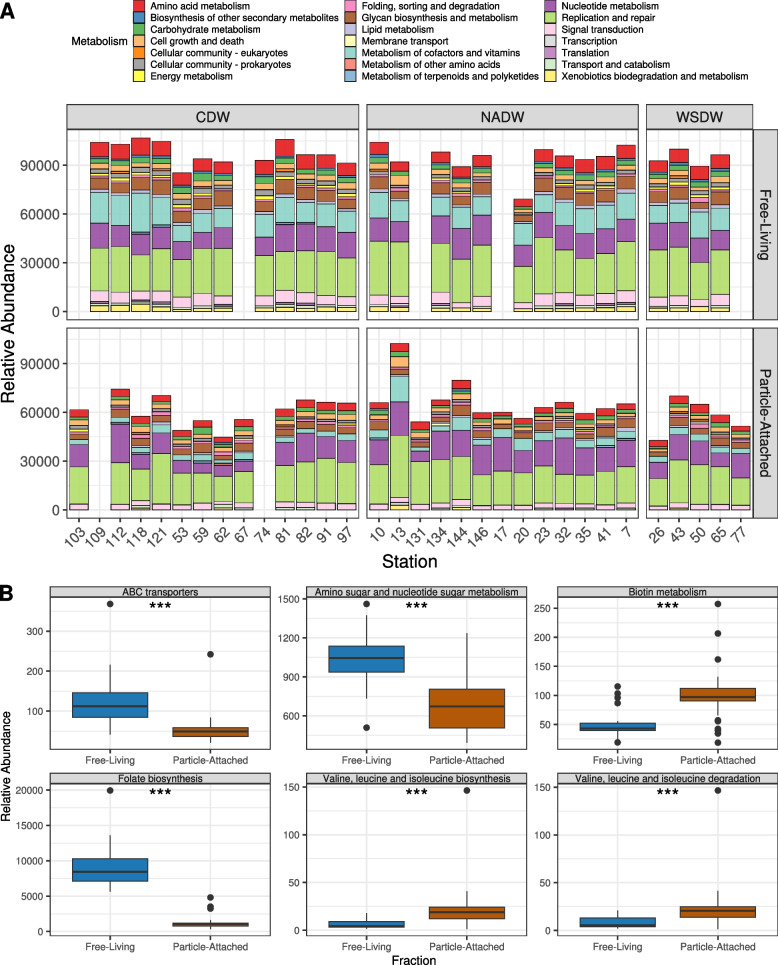
Differences in viral community functional composition between free-living and particle-attached samples. **A** Auxiliary metabolic gene KEGG module abundances across samples. Sampling stations are sorted from left to right by increasing oxygen concentrations. **B** Boxplots depicting the differences in pathway abundances between free-living and particle-attached fractions. Boxes depict the median, the first and third quartiles. Whiskers extend to 1.5 of the interquartile ranges. Outliers are represented as dots above or below whiskers. The triple asterisks indicate *p*-values < 0.001 obtained with the Mann-Whitney test. Sample MP0262 was excluded from this analysis as it displayed extremely high RPKM values, not in line with those observed in any other samples

The estimated abundances of viral-encoded KEGG metabolisms (calculated by grouping the relative abundances of individual KOs) were higher among the FL samples (Fig. 3A, Tables S7), although some pathways (Fig. 3B and Table S6), and specific KOs (Table S5), were significantly more abundant among PA samples (Table S10). The metabolism of cofactors and vitamins was the main AMG category responsible for the differences in relative abundances between FL and PA fractions at the “metabolism” level, as it was more abundant among the former. The aforementioned findings corroborate the observation that vitamin biosynthesis genes are enriched in FL microbial metagenomes compared to PA metagenomes obtained from the same Malaspina samples [15]⁠ and indicates that this pattern also extends to the viral fraction.

Regarding specific metabolic pathways, AMGs involved in ABC transporters; amino sugar and nucleotide sugar metabolism, and folate biosynthesis enzymes, were more abundant in FL samples (corrected *p*-value ≤ 0.05, Fig. 3B and Table S6). Concurrently, biotin metabolism, and the biosynthesis and degradation pathways of valine, leucine, and isoleucine, was relatively more abundant among PA samples (corrected *p* ≤ 0.05, Fig. 3B). These results are in line with previous evaluations of epipelagic viral community AMG content. Differences between functional composition of AMGs in FL and PA metagenomes from epipelagic samples were not statistically significant, although the differences in expression profiles of the same set of AMGs in metatranscriptomes were [20]. These differences in the associations between AMGs and environmental parameters of FL and PA lifestyles likely emerge as a consequence of the different constraints faced by bacteria and archaea in the epipelagic compared to those in the bathypelagic, as demonstrated by the extensive differences in AMG diversity and composition reported for these two habitats [21, 22, 37], and the evidence that epipelagic and bathypelagic microbial communities are structured and regulated by different sets of environmental parameters [3, 14, 15, 23, 24, 36, 38].

Based on these observations, we propose that in bathypelagic FL communities, which have less access to labile organic matter, viruses more often encode AMGs, manipulating host vitamin and nucleotide metabolism, as well as enhancing nutrient uptake through ABC transporters. These changes to host metabolism contribute towards successful production of viral progeny during infection among FL communities. Meanwhile, in PA communities, where more labile organic matter is available, viruses have less necessity to manipulate host metabolism during infection, but they still benefit from doing so by altering specific pathways of amino acid and vitamin metabolism.

**Fig. 4:**
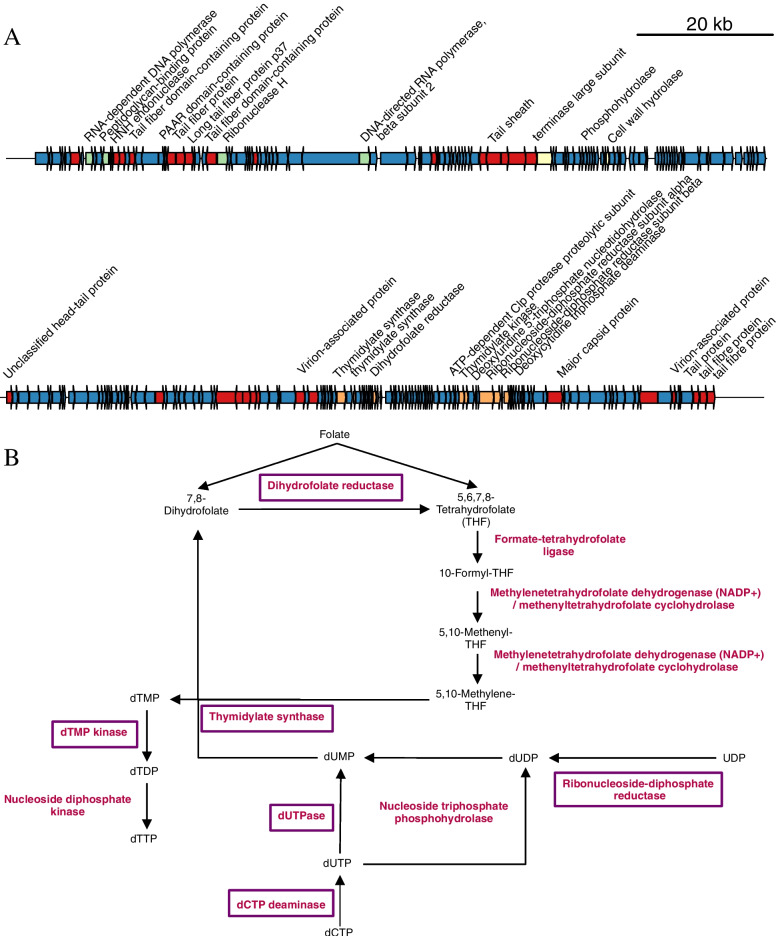
Genomic composition and AMG content of sequence Malaspina_Vir_6045. **A** Genomic map depicting the protein-encoding genes (depicted as arrows) identified in the viral scaffold. Genes are colour coded according to their functional annotation within the five categories: structural genes (red), genome replication genes (green), packaging and lysis genes (yellow), auxiliary metabolic genes (orange), and others (blue). For simplicity, for those genes annotated simply as "viral strucutural protein", the annotation text was omitted. **B** Proposed scheme illustrating the metabolic reactions within the folate and nucleotide pathways of the host which are under the influence of viral AMGs encoded in the genomic sequence of Malaspina_Vir_6045. Enzyme names are depicted in red; those genes encoded in the viral genome are enclosed by purple rectangles

Out of the 309 AMGs involved in the metabolism of cofactors and vitamins, only 54 were derived from viral scaffolds with a predicted host, which most often were Alphaproteobacteria (20) and Gammaproteobacteria (14). We investigated viral genomes encoding such genes in further detail, to determine the specific mechanisms by which viruses could modulate host vitamin metabolism in the bathypelagic. A total of 14 viral scaffolds had AMGs encoding for a dihydrofolate reductase (DHFR). This enzyme catalyses multiple reactions, including the conversion of folate (vitamin B9) and 7,8-dihydrofolate to 5,6,7,8-tetrahydrofolate (THF). Only three of the viral scaffolds encoding DHFR could reliably be assigned to a host (*p* ≤ 2.384e-14), one to Gammaproteobacteria and two to Marinisomatota. Among the 14 scaffolds, Malaspina_Vir_6045 was the longest (214 Kbp). This sequence was derived from a viral genome estimated to be 79% complete and classified as a member of the family Myoviridae. Host prediction would have associated this viral genome to a member of the class Gammaproteobacteria, yet this prediction did not pass our stringent threshold (*p* = 3.6e-04). Aside from DHFR, scaffold Malaspina_Vir_6045 was the only one that encoded multiple other AMGs, which gave us detailed insights about the potential of this virus to interfere with host metabolism (Fig. 4A). This viral genome encoded five AMGs involved in dTMP biosynthesis, namely ribonucleoside-diphosphate reductase (subunits alpha and beta), dCTP deaminase, dUTPase, thymidylate synthase, and dTMP kinase. Thymidylate synthase catalyses the conversion of dUMP into dTMP using 5,10-methylene-tetrahydrofolate as a co-substrate, which is subsequently converted to dTDP by dTMP kinase, and finally to dTTP by a nucleoside diphosphate kinase (Fig. 4B). The occurrence of these genes in a single genome suggests that this virus has the potential to enhance THF biosynthesis during infection, and redirect it to the synthesis of deoxynucleotides to be used in viral genome replication.

The relative abundances of Malaspina_Vir_6045 ranged between 0 and 223 RPKM, and the mean abundance was 10 RPKM, which falls into the 69th percentile when considering the relative abundances of all scaffolds across all samples. Relative abundances for Malaspina_Vir_6045 were higher among PA samples, specifically those from NADW. When considering DHFR-encoding viral scaffolds together, the opposite pattern was observed, as these had a mean abundance of 61 RPKM among FL samples and 23 RPKM among PA samples (Fig. S1). The current results provide evidence that the viruses play a role in the bathypelagic vitamin budget. DHFR genes have previously been reported in the genomes of marine bacteriophages [39]⁠. Yet, to our knowledge, this is the first time this gene is reported in bathypelagic viruses, in association with other genes directly involved in nucleotide metabolism, and with differential abundances between free-living and particle-attached fractions or water masses. Thus, it is possible that the abundance of DHFR-encoding viruses is partially regulated by the exchange of vitamins between their free-living and particle-attached hosts.

We interpreted these patterns as an indication that AMGs, especially those involved in vitamin metabolism, are proportionally more prevalent among FL viral communities compared to their PA counterparts. Nevertheless, it is possible that the higher relative abundances of putative AMGs observed among FL samples was influenced by the fact that KEGG annotation was more efficient for proteins derived from FL than PA viruses. Namely, 940 viral scaffolds were significantly more abundant among FL metagenomes (Bonferroni corrected *p*-value of Mann-Whitney test ≤ 0.05), while 514 viral scaffolds were significantly more abundant among PA metagenomes (Bonferroni corrected *p*-value of Mann-Whitney test ≤ 0.05). Among the protein-encoding genes predicted from the FL-enriched scaffolds, 15.2% were assigned a KEGG KO, while only 5.9% of the protein-encoded genes predicted from the PA-enriched scaffolds were assigned KEGG KOs. Thus, it is possible that the higher abundance of most functional categories observed among FL samples was driven by differences in the proportion of unknown genes among the two size fractions.

### Viral community abundance, AMG diversity, and composition are associated with water mass age

**Fig. 5:**
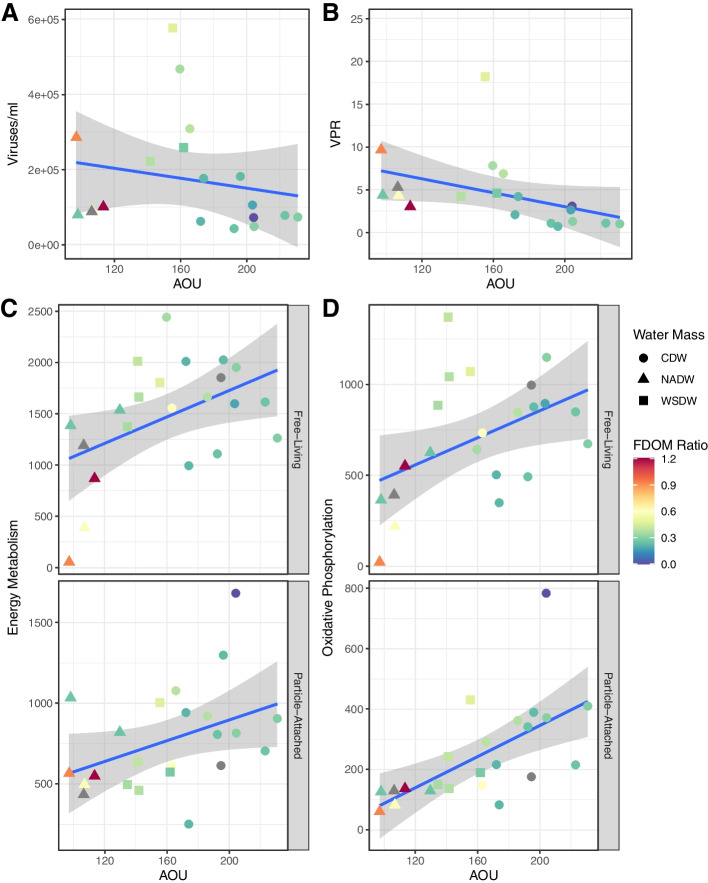
Associations between deep ocean viral communities and AOU. Scatter plots depict the association between AOU (*x*-axis) and the tested variable (*y*-axis). The blue line depicts the best fit for linear regression models with shaded areas depicting the standard error. **A** Association between viral absolute abundance and AOU. **B** Association between virus-to-prokaryote ratio (VPR) and AOU. **C** Association between relative abundance of energy metabolism AMGs and AOU in FL and PA samples. **D** Association between relative abundance of oxidative phosphorylation AMGs and AOU in FL and PA samples. Sample MP0262 was excluded from this analysis as it displayed extremely high RPKM values, not in line with those observed in any other samples

Viral particle abundances and virus-to-prokaryote ratios, quantified by flow cytometry, were negatively correlated with AOU (Fig. 5 A and B, Spearman correlation coefficient = −0.45, *p* < 0.05 and = −0.72, *p* < 0.001, respectively). Likewise, a significant positive correlation was observed between AOU and the relative abundance of the AMGs related to energy metabolism (Fig. 5C, Spearman correlation coefficient = −0.34, *p* < 0.05) and between AOU and AMGs of the oxidative phosphorylation pathway (Spearman correlation coefficient = −0.43, *p* < 0.005, Table S11, Fig. 5D).

The association patterns between AOU and the absolute abundances of viruses, as well as the relative abundances of the AMGs encoded within them, suggest that the composition and functioning of viral communities are influenced by water mass age. Even among the water masses with highest AOU values, it is unlikely that the microbial communities therein are subjected to a limited oxygen supply. Therefore, the observed associations with AOU are likely linked to the differences in organic matter content among water masses of different ages. The optical properties of dissolved organic matter can be used as a tracer of biochemical processes [40]⁠, distinguishing between humic-like (recalcitrant) versus amino-acid-like (labile) fluorescence. Thus, the ratio between labile and recalcitrant fluorescence in a sample (hereafter FDOM ratio) may be used as an indicator of the availability of labile resources [15]⁠. Younger water masses have higher FDOM ratio and lower AOU, as a water masses age; microbial activity consumes labile organic compounds and oxygen, leading to higher AOU and lower FDOM ratio.

Differences in the FDOM ratio among water masses were significant (Mann-Whitney test *p* < 0.05) when comparing CDW against NADW, and CDW against WSDW, but not when NADW and WSDW were compared (*p* = 0.49). Furthermore, values of FDOM ratio had positive and significant correlations with viral abundances (Spearman correlation coefficient = 0.48, *p* < 0.05) and virus-to-prokaryote ratios (Spearman correlation coefficient = 0.53, *p* < 0.05). The relative abundance of energy metabolism AMGs was also negatively correlated to the FDOM ratio (Spearman correlation coefficient = −0.32, *p* < 0.05). Analogous to the dichotomy between FL and PA lifestyles, in younger water masses with higher FDOM ratio, energy is readily available to the microbial hosts through the use of labile organic compounds. Conversely, in older water masses with lower FDOM ratio, the hosts need to be more efficient at utilising recalcitrant organic compounds as the main energy source.

**Fig. 6:**
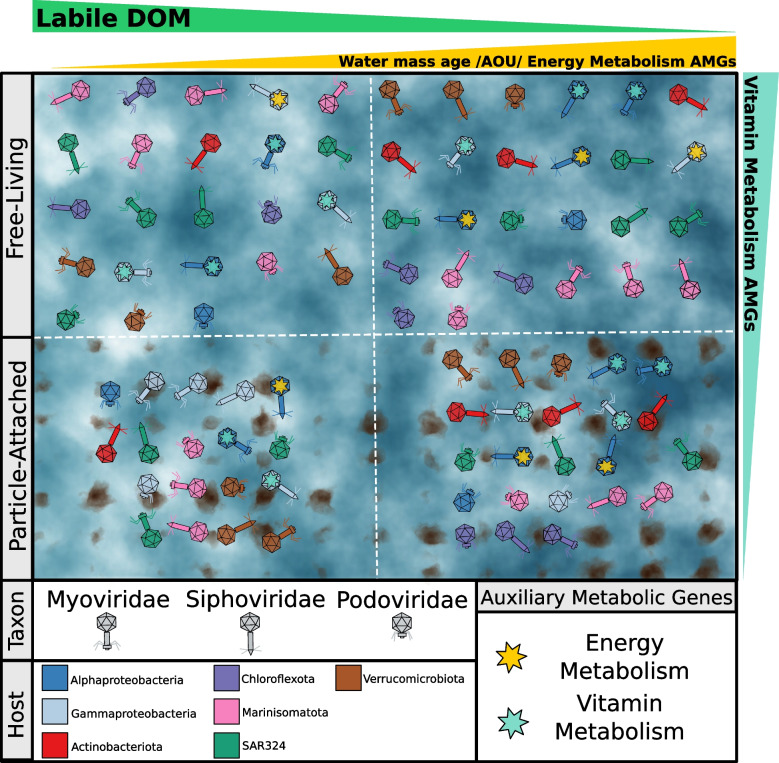
Conceptual model illustrating the changes in viral community between FL and PA and throughout the AOU and FDOM gradients. The most abundant viral families of tailed viruses (Myoviridae, Siphoviridae, and Podoviridae) are depicted according to their morphology. Virus colouring is indicative of a predicted host. Auxiliary metabolic genes are depicted by coloured stars inside viral particles

We posit a mechanism to explain the higher relative abundances of AMGs involved in the energy metabolism and oxidative phosphorylation observed among older water masses (Fig. 6). Previous findings showed that the genomes of the hosts that thrive within older water masses encode more genes associated with energy metabolism and oxidative phosphorylation, as a mechanism to more efficiently harness energy from the limited supply of organic matter [15]⁠. Thus, the higher relative abundance of AMGs involved in these pathways among the viral genomes could reflect the uptake of metabolic genes from the host genomes. Those viruses that encode such AMGs could have a selective advantage, as they would increase the efficiency of the energy metabolism of their hosts during infection, leading to more viral progeny, and higher relative abundance within the viral community.

## Conclusions

Our findings provide insights about deep ocean viruses, one of the least explored biological entities to date. The data corroborates our hypotheses that (1) free-living and particle-attached fractions have different viral community compositions; (2) environmental factors, mainly those related to water mass age, control the abundance, diversity, and AMG repertoire of both free-living and particle-attached viral communities; (3) particle-attached communities are enriched in viruses that target copiotrophic bacteria, while free-living communities are enriched in viruses that target oligotrophic bacteria; and (4) AMGs from metabolic pathways associated with energy metabolism are enriched in older water masses characterised by lower concentrations of labile organic matter. Our findings lay a solid foundation to the understanding of the factors that structure and influence the functioning of bathypelagic viral communities, by providing a genome, taxonomy, and host-resolved dataset of viruses that includes novel AMG combinations.

## Methods

### Sample collection and environmental parameters

These procedures have previously been described in detail [14]⁠. Briefly, samples were collected as part of the 2010 Malaspina circumnavigation expedition (http://www.expedicionmalaspina.es), which covered both tropical and subtropical regions of the global ocean (Table S1). Measurements of water temperature, salinity, and oxygen concentrations were taken in situ [24]. Salinity ranged from 34.64 to 34.91 (median = 34.7, *SD* = 0.09); temperature ranged from 0.58 to 2.25 °C (median = 1.5, *SD* = 0.48).

Apparent oxygen utilisation (AOU) is the parameter used to estimate water age. It is calculated as the difference between oxygen solubility in a water mass and its measured oxygen concentration, integrating all respiratory processes since the last contact of the water mass with the atmosphere. Oxygen solubility is determined by pressure, water temperature, and salinity. Older water masses have lower oxygen concentrations and higher AOU and vice versa. AOU ranged from 97 to 231 μmol/kg (median = 163, *SD* = 39). There were significant associations between salinity, temperature, and AOU (*p* < 0.01, Pearson product-moment correlation).

Water samples were retrieved to characterise the fluorescent dissolved organic matter (FDOM) content of the water masses from which the metagenomes were obtained [26, 41]⁠. The FDOM composition is characterised by a pair of recalcitrant humic-like compounds (C1 and C2), and a pair of comparatively more labile compounds (C3 and C4), which are commonly attributed to the amino acids tryptophan and tyrosine respectively [26, 41]⁠. The FDOM ratio was calculated as (C3 + C4)/(C1 + C2). This variable is a proxy for the proportion of labile compounds in the dissolved organic matter pool⁠.

### Metagenome and viral genome analysis

For each sample, 120 l of seawater was sequentially filtered through 200–20-μm meshes to remove larger eukaryote cells. Next, size fractioning was used to separate the free-living (0.2–0.8 μm) and particle-attached (0.8–20 μm) fractions. Filtered samples were flash frozen in liquid nitrogen until DNA extraction which was performed through the phenol-chloroform method. DNA was sequenced at the DOE’s Joint Genome Institute (JGI) in an Illumina HiSeq 2000 platform. Metagenome read quality control was performed following JGI’s standard operational procedure. Briefly, raw sequences shorter than 150 bp were removed and then trimmed in order to remove quality regions and trailing ambiguous base calls. Further quality trimming was carried out by keeping the longest subsequence above a quality threshold Q13 in Lucy 1.20 [42] and discarding sequences with more than 5′ N’s or shorter than 150 bp. Low complexity regions were filtered and removed with dustmasker 1.0.0 [43]. Average read length after QC was 132 bp (SD + −2.6). Post-QC reads from metagenomes were co-assembled using Megahit v1.2.8 with options −presets meta-large −min-contig-len 2000 [37]⁠. Scaffolds were de-replicated with cd-hit-est (v4.8.1 compiled for long sequence support; MAX_SEQ = 10,000,000, with options −c 0.95 −n 10 −G 0 −aS 0.95 −d 0) [44]⁠.

All of the following analyses were performed with default parameters unless otherwise stated. All the assembled scaffolds derived from the co-assembly of metagenomes were processed through VIBRANT v1.2.1 [27]⁠ to identify viral sequences. The quality of the obtained viral genomes and genome fragments was assessed with CheckV v0.7.0 [28]. Taxonomic classification of viral sequences was achieved through VPF-Class version dd88a54 [29]. Viral host predictions were performed using PHIST version ed2a1e6 [30]⁠, the previously described set of metagenome assembled genomes from the Malaspina dataset [14]⁠, and were used as putative hosts. For the PHIST analysis, only predictions with a maximum *e*-value of 2.384e-14 were considered, which yields approximately 85% class level prediction accuracy.

Coding DNA sequences (CDS) in viral genomes were identified with Prodigal v2.6.3 [45]⁠. Viral CDS were queried against a large database containing protein sequences derived from approximately 4.6 million viral genomes and genome fragments derived from the latest release of IMG/VR [46]⁠ and complemented with sequences from multiple ecosystems. Protein searches were performed using MMSeqs2 [47]. Next, the pairwise similarity scores were calculated between query and reference genomes based on the number of matched CDS, percentage of matched CDS, and average amino acid identity (AAI). To exclude spurious similarities, the following thresholds were applied: a minimum of 3 CDS matches, covering at least 30% of all CDS in the query scaffold, and yielding a minimum of 30% AAI. CDS were queried against three databases for functional taxonomic annotation: (1) UniRef100 using DIAMOND version 2.0.7 [48]⁠, (2) KOFam using Hmmscan version 3.3[49]⁠, and (3) Pfam using Hmmscan as well. For all searches, only hits that displayed a bit score ≥ 50, and *e*-value ≤ 10^−5^ were considered for subsequent analysis

### Viral community composition analysis

Post-QC reads from the metagenomes were queried against the database of obtained viral genomes using Bowtie v2.3.4.1 [50] in sensitive local mode. On average, 328,595 (SD ± 469,646) reads per metagenome mapped to the viral scaffolds, representing an average of 2.98 % (SD ± 3.6%) of the post-QC reads from each metagenome. Genome abundances were calculated as reads per kilobase per million total sequences (RPKM). Viral genome abundances were grouped by viral families or predicted host taxon as the sum of RPKM values of all the viruses in each group⁠. Similarly, abundances of KEGG KOs were calculated as the sum of RPKM values of all genomes encoding a given KO. Finally, the abundances of KEGG pathways and metabolisms were calculated as the sum of all genomes encoding a given KO associated with a metabolism/pathway (Table S5). To avoid overestimating abundances for KOs that belong to multiple metabolisms/pathways, the abundance of each KO was divided by the number of metabolisms/pathways the KO was assigned before calculating sums (Tables S6 and S7).

### Statistical analysis

Sample community composition analyses were performed based on calculated RPKM values of viral scaffolds. We performed a distance-based RDA (dbRDA) analysis as follows: Bray-Curtis distances between samples were calculated using the relative abundance (RPKM) values of viral scaffolds as input, using the dbrda function from the Vegan package [51]⁠. These distances were ordinated in a bidimensional space constrained by AOU and size fraction. Permutational multivariate analysis of variance (PERMANOVA) was conducted with 1000 permutations and Bray-Curtis distances. In addition, we performed Mantel tests (using Spearman’s rank correlation and 999 permutations) to detect associations between environmental parameters and community composition. Euclidean distances among samples were calculated individually for latitude, depth, temperature, salinity, and O_2_ concentration. Differences in variable relative abundances (i.e. taxa, predicted hosts, or functions) between free-living and particle-attached fractions were evaluated with the Mann-Whitney test. Associations between environmental and metagenome variables were assessed by calculating either Pearson or Spearman correlation scores. For both correlation and Mann-Whitney tests, associations with a *p*-value ≤ 0.05 were considered significant. Multiple testing correction was performed through the Bonferroni method. All analyses were carried out in R v4.0.0 [52].

### Supplementary Information

**Additional file 1:** **Table S1.** Metadata of the collected samples and metagenomes. **Table S2.** Sequence data of the obtained viral genomes and genome fragments. **Table S3.** Functional and taxonomic annotation of the CDS encoded in the viral genomes and genome fragments. **Table S4.** Relative abundances of viral viral genomes and genome fragments across 58 metagenomes calculated as RPKM. **Table S5.** Relative abundances of viral KEGG KOs across 58 metagenomes calculated based on RPKM of individual viral viral genomes and genome fragments. **Table S6.** Relative abundances of viral KEGG pathways across 58 metagenomes calculated based on RPKM of individualviral genomes and genome fragments. **Table S7.** Relative abundances of viral KEGG metabolism across 58 metagenomes calculated based on RPKM of individual viral genomes and genome fragments. **Table S8.** Relative abundances of viruses grouped according to family level taxonomic affiliation based on RPKM of individual viral genomes and genome fragments. **Table S9.** Relative abundances of viruses grouped according to predicted host phylum (or class for Proteobacteria) taxonomic affiliation based on RPKM of individual viral scaffolds. **Table S10.** Results of Mann-Whitney tests used to compare the relative abundances of: viruses grouped according to predicted host phylum (or class for Proteobacteria), family level taxonomic affiliation, viral KEGG pathways, and viral KEGG metabolisms. **Table S11.** Results of Pearson and Spearman correlation analyses used to quantify the degrees of association between environmental parameters and the relative abundances of: viruses grouped according to predicted host phylum (or class for Proteobacteria), family level taxonomic affiliation, viral KEGG pathways, and viral KEGG metabolisms.

**Additional file 2:** **Fig. S1.** Relative abundance patterns of viral scaffolds encoding DHFR genes. A) Stacked bar plots depicting the RPKM abundances (y-axis) of viral scaffolds across samples (x-axis), separated by free-living and particle-attached samples (panels). Sampling stations are sorted from left to right by increasing oxygen concentrations. B) Box plots depicting the differences in DHFR encoding scaffold abundances between free-living and particle-attached fractions. Boxes depict the median, the first and third quartiles. Whiskers extend to 1.5 of the interquartile ranges. Outliers are represented as dots above or below whiskers. The p-values of each comparison obtained with the Mann-Whitney test are depicted above bars.

## Data Availability

Viral scaffolds were deposited in ENA under project ID PRJEB40454. The code used to perform genomic sequence analysis is available at https://github.com/felipehcoutinho/virathon.
