# Supplementary figures and images for: Water mass age structures the auxiliary metabolic gene content of free-living and particle-attached deep ocean viral communities
Source: Microbiome. 2023 May 27;11:118. doi: 10.1186/s40168-023-01547-5 (PMC10224230; doi:10.1186/s40168-023-01547-5)

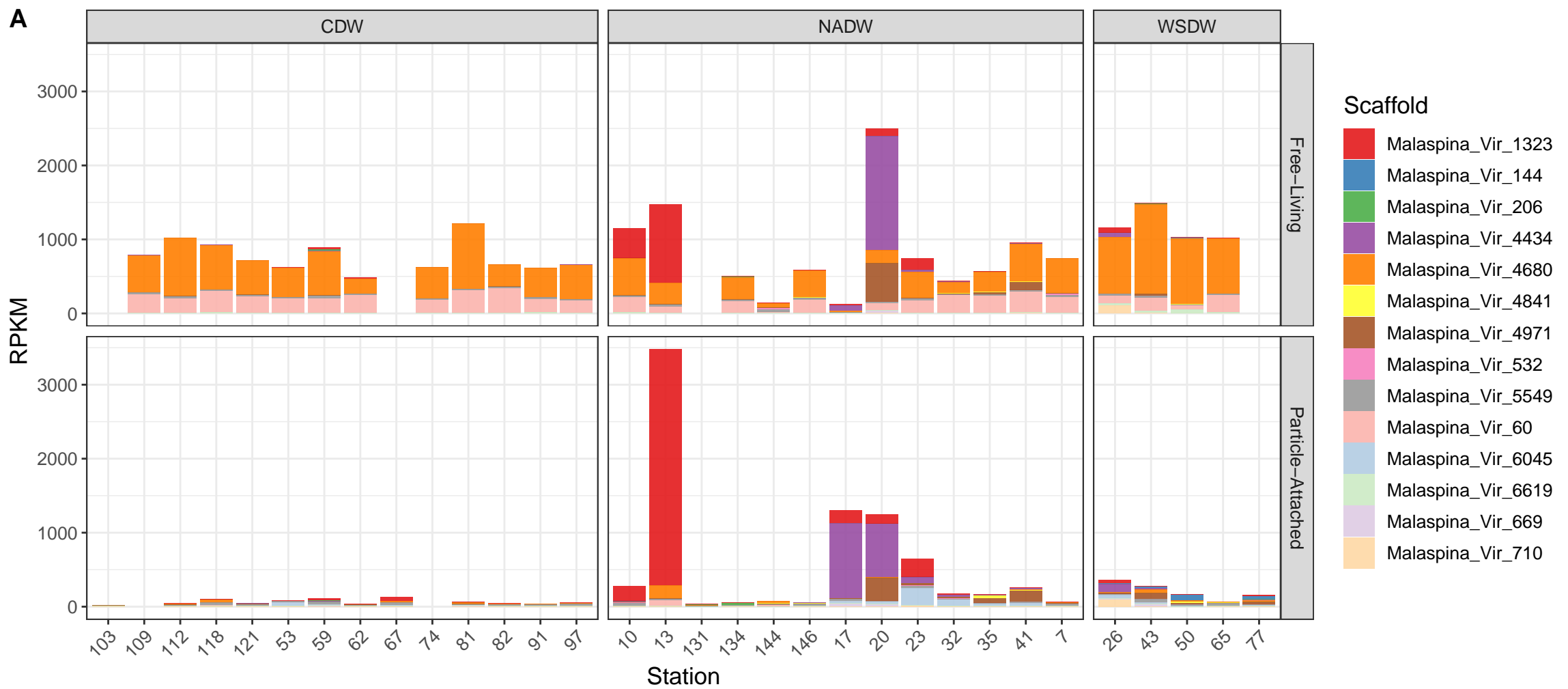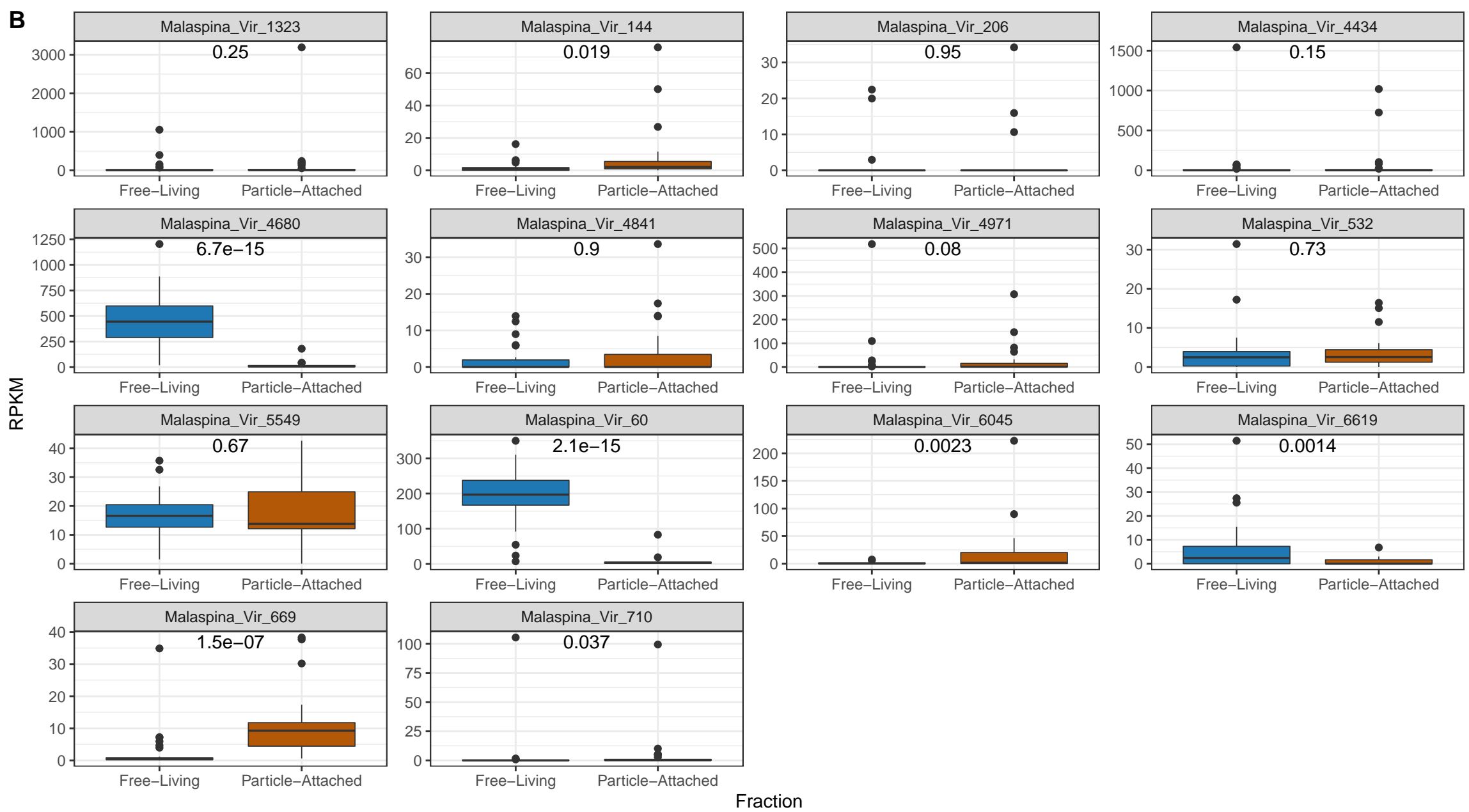

Supplement: Supplementary file 3 — Additional file 2: Fig. S1. Relative abundance patterns of viral scaffolds encoding DHFR genes. A) Stacked bar plots depicting the RPKM abundances (y-axis) of viral scaffolds across samples (x-axis), separated by free-living and particle-attached samples (panels). Sampling stations are sorted from left to right by increasing oxygen concentrations. B) Box plots depicting the differences in DHFR encoding scaffold abundances between free-living and particle-attached fractions. Boxes depict the median, the first and third quartiles. Whiskers extend to 1.5 of the interquartile ranges. Outliers are represented as dots above or below whiskers. The p-values of each comparison obtained with the Mann-Whitney test are depicted above bars. [file 40168_2023_1547_MOESM2_ESM.pdf]
